# Identification of immune cells and mRNA associated with prognosis of gastric cancer

**DOI:** 10.1186/s12885-020-6702-1

**Published:** 2020-03-12

**Authors:** Mingming Wang, Zedong Li, Yu Peng, Jianyu Fang, Tao Fang, Jiajia Wu, Jun Zhou

**Affiliations:** 1 Department of Minimally Invasive Surgery, The Second Xiangya Hospital, Central South University, Changsha, 410011 Hunan China; 2 Department of Nursing, The Second Xiangya Hospital, Central South University, Changsha, Hunan China; 3 Department of General Surgery, The First Affiliated Hospital of Gannan Medical University, Ganzhou, Jiangxi China; 4 Department of General Surgery, The Second Affiliated Hospital of Nanjing Medical University, Nanjing, Jiangsu China

**Keywords:** Gastric cancer, Immune infiltration, SUPV3L1, SLC22A17

## Abstract

**Background:**

The clinical success demonstrates the enormous potential of immunotherapy in cancer treatment.

**Methods:**

This article presented research linking gastric cancer to immune cells, based on RNA-seq data of Stomach adenocarcinoma (STAD) and gene expression profile of GSE84437, 24 kinds of tumor-infiltrating immune cells were quantified by single-sample gene set enrichment analysis.

**Results:**

Th2 cells, T helper cells, and Mast cells were identified as prognostic immune cells in both TCGA and GEO groups. Then SUPV3L1 and SLC22A17 were identified as hub genes which may affect immune cell infiltration by correlation analysis. Survival analysis further proved that hub genes and prognostic immune cells are associated with the prognosis of gastric cancer. In gastrointestinal tumors, hub genes and prognostic immune cells also found differences in non-tumor and tumor tissues.

**Conclusions:**

We found that three immune cells infiltration are associated with the prognosis of gastric cancer and further identify two hub genes. These two key genes may affect immune cell infiltration, result in the different prognosis of patients.

## Background

Many experimental and theoretical studies indicate that most solid tumors are associated with immune infiltrate, as early as 15 years ago, immune response within colorectal cancers are associated with early metastatic invasion and survival were introduced by Franck Pagès et al. [1]. In some digestive system neoplasms, immune cells may inhibit tumor progression, T cell infiltration is closely related to the patient prognosis of colorectal cancer, and types of lymphocytic infiltration, density, and intratumoral location may better predict prognosis than TNM or Duke’s classification [1, 2]. With the deepening of research on immune-related mechanisms, immunotherapy and application of immune-checkpoint inhibitors make it possible to effective treatment or even cure several malignancies previously untreated [3, 4]. However, the role and type of tumor-infiltrating immune cells in the prognosis of gastric cancer is unknown, identification of immune cells associated with tumor prognosis and new immune-related therapeutic targets in gastric cancer is the urgent need to solve practical problems.

Tumors are composed of many types of cells, the main part of which is a large number of malignant cells. Tumor-infiltrating immune cells are also one of the types that play an important role [5, 6], for instance, T cells are one step in the elimination of cancer, they can specifically recognize and kill tumor cells and manage the delicate balance between the recognition of nonself and the prevention of autoimmunity [7]. Quantification of infiltrating immune cells in tumors may untie the role of immune cells in tumor progression and provide a new direction for immunotherapy. Heretofore, immune infiltration has been primarily studied by immunohistochemistry, immunofluorescence and flow cytometry. But with the widely used of next-generation sequencing (NGS) technologies, tumor RNA-Seq data can be obtained from the database, such as the cancer genome atlas (TCGA) and Gene Expression Omnibus (GEO). Based on a set of immune-specific marker genes, MCP-counter, single-sample Gene set enrichment analysis (ssGSEA), CIBERSORT and other computational approaches can be used to quantify tumor-infiltrating immune cells from RNA sequencing data [8–10]. Therefore, we attempted to quantify tumor-infiltrating immune cells across human healthy tissues and tumors based on the ssGSEA method and identify genes associated with prognosis-related immune cells.

## Materials and methods

### Data collection

We download gene expression data, somatic mutation data and clinical data of stomach adenocarcinoma (STAD) from the cancer genome atlas (TCGA) database by TCGAbiolinks and maftools packages in R (3.5.1, [11, 12]). In order to verify the results of the study in the TCGA data, gene expression profile and clinical data in GSE84437 were downloaded from the Gene Expression Omnibus (GEO) database. In the TCGA dataset, samples with death reason of other malignancy, other and non-malignant disease, sample type is not “Primary Tumor”, and samples with incomplete overall survival information were excluded, total 360 samples were included finally. The GEO dataset included 433 gastric cancer tissues. For further investigate the underlying mechanisms in digestive system tumors, gene transcripts per million (TPM) data of Pan-cancer in TCGA and normal tissues in genotype tissue expression (GTEx) database were downloaded from the UCSC Xena database, which processed by the TOIL process, free of computational batch effects. All analyses and plots are done by R (3.6.0).

### Data preprocessing and quantification of immune cells

Gastric cancer patients died of non-malignant disease and other malignancies were excluded, and samples with complete survival data were included. According to Gabriela Bindea et al., we obtained the marker genes of 24 immune cells, including aDC, B cells, CD8 T cells, Cytotoxic cells, DC, Eosinophils, iDC, Macrophages, Mast cells, Neutrophils, NK CD56 bright cells, NK CD56 dim cells, NK cells, pDC, T cells, T helper cells, Tcm, Tem, TFH, Tgd, Th1 cells, Th17 cells, Th2 cells and TReg [10] . Then, based on the gene expression data and marker genes, infiltrating immune cells were quantified by ssGSEA.

### Survival analysis

The association between immune cells and overall survival was carried out using univariate Cox regression, immune cells with statistically significant(*P* < 0.05) in both groups be considered as effects of prognosis. For further evaluate the impact of the immune cells with statistically significant, patients were divided into 2 groups according to the method of best separation in “survminer” R package, then, overall survival were analyzed by “survival” R package. Kaplan Meier-plotter (KM plotter, http://kmplot.com/analysis/) could assess the effect of hub genes on survival [13] . The hazard ratio (HR) with 95% confidence intervals and log rank *P* value were calculated and displayed on the plot.

### Hub genes identification and validation

Hub genes were several genes that are related to immune cells. The methods of Pearson correlation coefficient and Spearman’s rank correlation coefficient were used for calculation of the correlation between gene expression and immune cells, genes with *P* < 0.01 and correlation> 0.3 were included in the follow-up study. To further study genes associated with immune cells. Genes in the intersection of all groups (genes associated with Th2cells, T helper cells, and mast cells in the TCGA and the GEO groups) were selected as hub genes. The method of survival analysis of hub genes is the same as the previous step.

### Assessment of tumor mutational burden

Data of tumor mutational burden were downloaded by “TCGAbiolinks” R package, “Maftools” R package was used to read the maf files and count the number of variants in each sample. We tried to analyze whether there are differences in tumor mutational burden (TMB) between the high and low expression of hub genes and prognostic immune cells. 322 samples with complete survival information, gene expression data and TMB were included. According to the method of best separation in “survminer” R package, patients were divided into groups of high and low, and the Wilcoxon test was used to identify differences of tumor mutational burden(*p* < 0.05).

### Differences in tumor and normal tissues

Gastric cancer is one of digestive system tumor, we further compare the differences of immune cells and hub gene expression between digestive tumors and normal tissue. Gene transcripts per million (TPM) of digestive system normal and tumors tissues were downloaded from UCSC Xena (https://xenabrowser.net/datapages/), Normal tissue data is from Genotype tissue expression (GTEx) database, tumor tissue data is from TCGA database, and infiltrating immune cells were quantified by ssGSEA.

### Functional annotation of hub genes

Gene counts of TCGA-STAD were downloaded by “TCGAbiolinks” R package, patients were divided into 2 groups according to the expression of hub genes by method of best separation. Then, differentially expressed genes (DEGs) screened between the high and low group, gene set enrichment analysis (GSEA) [14] and enrichment analysis were performed with “clusterProfiler” package in R [15]. We use “GOSemSim” package to calculate the similarity between Gene Ontology (GO) terms and then plot it with “ggtree” package.

## Results

### Immune cells identification and survival analysis

**Fig. 1:**
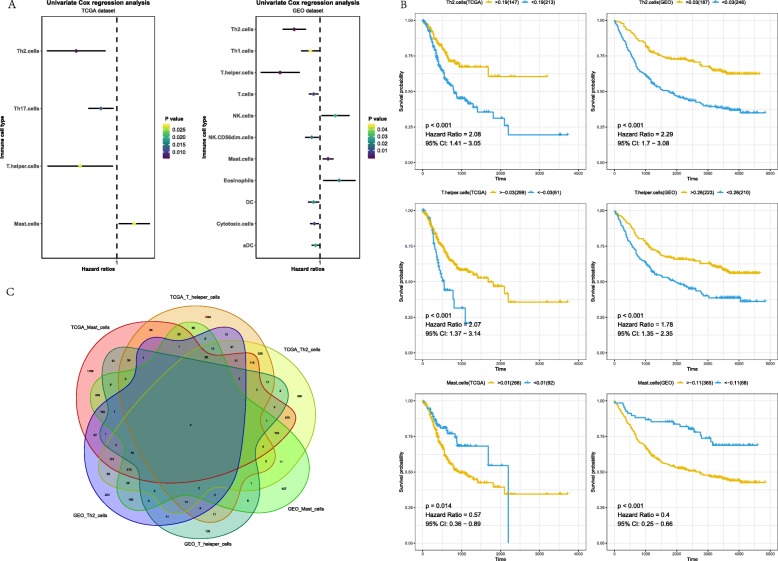
Identification of immune cells and related genes associated with prognosis in patients with gastric cancer. **a** The left side of the dotted line represents HR < 1, which is a protective factor, and the right side represents HR > 1, which is a risk factor. **b** Survival analyses on selected immune cells in the TCGA and GEO data set. Survival curves for patients in different groups. Yellow lines represent high infiltration of immune cells, while blue lines represent low infiltration of immune cells. C: There were 2 genes in the intersection of 6 gene sets

After quantification of infiltrating immune cells, univariate Cox regression was used to screen immune cells that affect prognosis. Results of TCGA and GEO datasets were shown in Fig. 1a. Infiltration of Th2 cells, T helper cells and Mast cells (*P* < 0.05) related to the survival of patients with gastric cancer in two datasets, and the three immune cells showed the same effect. Th2 cells and T helper cells were protective factors, and Mast cells were risk factors. The survival plot based on the best separation of high and low infiltration of each immune cell in TCGA and GEO datasets. As shown in Fig. 1b, patients with higher each protective immune cells showed a significantly higher overall survival rate, patients with higher risk immune cell showed a significantly lower overall survival rate.

### Hub genes identification and validation

**Fig. 2:**
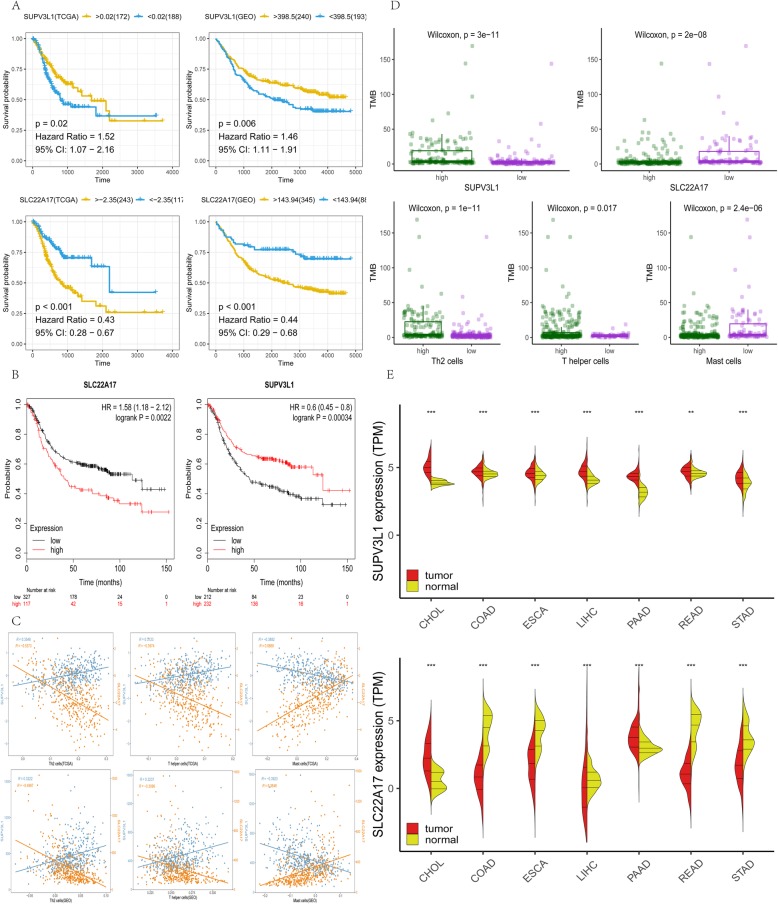
**a** Yellow lines represent a high expression of the gene, while blue lines represent a low expression of the gene. **b** Prognostic value of SUPV3L1 and SLC22A17 in gastric cancer patients were reconfirmed by Kaplan Meier-plotter .**c** Correlation between genes and immune cells. **d** Green represents high infiltration of immune cells, purple represents low infiltration of immune cells. **e** In cholangiocarcinoma (CHOL), Colon adenocarcinoma (COAD), Esophageal carcinoma (ESCA), Liver hepatocellular carcinoma (LIHC), Pancreatic adenocarcinoma (PAAD), Rectum adenocarcinoma (READ) and Stomach adenocarcinoma (STAD), expression of both genes shows significant differences in adjacent tissues and tumor tissues

To further clarify the regulatory relationship of mRNA and prognostic immune cells, methods of Pearson correlation coefficient and Spearman’s rank correlation coefficient were used to calculate the correlation between mRNA and prognostic immune cells. By two methods, genes under the threshold of *P* < 0.01 and correlation> 0.3 were selected. In TCGA group, 4844 genes which associated with Mast cells, 2160 genes which associated with T helper cells and 2984 genes which associated with Th2 cells were screened out, in GEO group, 2128 genes which associated with Mast cells, 372 genes which associated with T helper cells and 1590 genes which associated with Th2 cells were screened out. SUPV3L1 and SLC22A17 (Fig. 1c), they are considered as the hub genes that associated with infiltration of three prognostic immune cells. The survival plot based on the best separation of high and low expression of hub genes in TCGA and GEO datasets. Thus, SUPV3L1 expression was used to divide patients into SUPV3L1^high^ (172 samples) and SUPV3L1^low^(188 samples) groups and SUPV3L1 expression was used to divide patients into SLC22A17^high^ (243 samples) and SLC22A17^low^(117 samples) groups. As shown in ​Fig. 2a, patients with higher expression of SUPV3L1 showed significantly higher overall survival rate, patients with higher expression of SLC22A17 showed significantly lower overall survival rate. Prognostic value of SUPV3L1 and SLC22A17 in gastric cancer patients were reconfirmed by Kaplan Meier-plotter (KM plotter, http://kmplot.com/analysis/). It was found that expression of SLC22A17 (HR = 1.58 (1.18–2.12), *P* = 0.0022) was associated with worse overall survival (OS) for gastric cancer patients,expression of SUPV3L1 (HR = 0.6 (0.45–0.8) *P* = 0.00034) was associated with good overall survival (OS) for gastric cancer patients (Fig. 2b).

### Correlation between hub genes and prognostic immune cells

To show the correlation of hub genes and prognostic immune cells, we calculated the correlation by methods of Pearson correlation coefficient and plotted (Fig. 2c). SUPV3L1 was positively correlated with Th2 cells and T helper cells, and negatively correlated with Mast cells, SLC22A17 was exactly the opposite.

### Association with tumor mutational burden

Tumor mutational burden of gastric cancer in TCGA were downloaded by “TCGAbiolinks” R packages. According to hub genes expression and infiltration of prognostic immune cells, 322 samples were divided into groups of high and low. There was a significant difference (*P* < 0.05) in tumor mutational burden between patients in the high and low group, high expression of SUPV3L1 and infiltration of Th2 cells and T helper cells and low expression of SLC22A17 and infiltration of Mast cells coupled with a high mutational burden (Fig. 2d). It may indicate that a high mutational load coupled with high infiltration of Th2 cells and T helper cells and low infiltration of Mast cells, TMB-H (high tumor mutation load) patients produce more new antigens, and tumors are attacked by a large number of Th2 cells and T helper cells.

### Differences in tumor and normal tissues

**Fig. 3:**
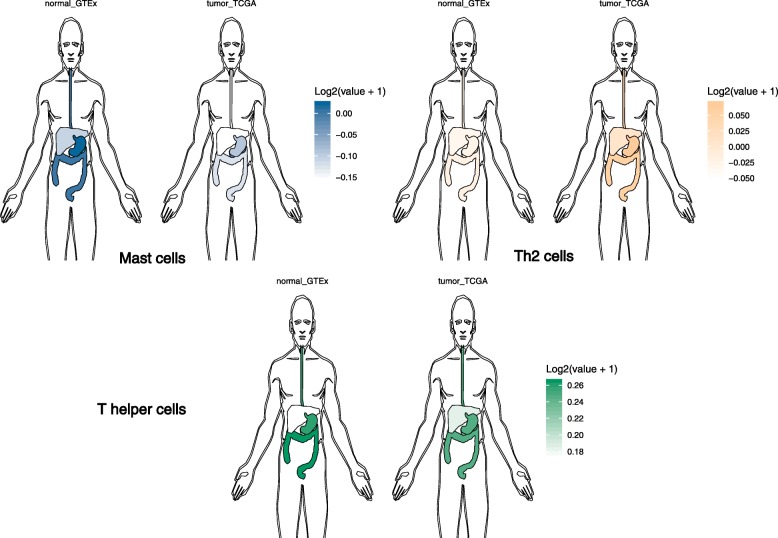
Normal_GTEx represents normal samples of the dataset from GTEx, tumor_TCGA represents tumor samples from the TCGA data set, the darker the color, the higher the degree of infiltration

Digestive system tumor compared with paracancer, there was a significant difference (*P* < 0.05) in the expression of SUPV3L1 and SLC22A17 (Fig. 2e). Through comparing with infiltration of prognostic immune cells in the tumor and normal tissues, we have summarized that Mast cells and Th2 cells in the digestive system normal tissues and tumor were different. Compared to the normal tissues, the population of Th2 cells in tumor had more, but Mast cells and T helper cells in the tumor were less than normal tissues, where liver seems different, Mast cells in normal liver tissue nearly no, and liver tumor tissue has a small amount of Mast cells infiltration (Fig. 3).

### Functional annotation of hub genes

**Fig. 4:**
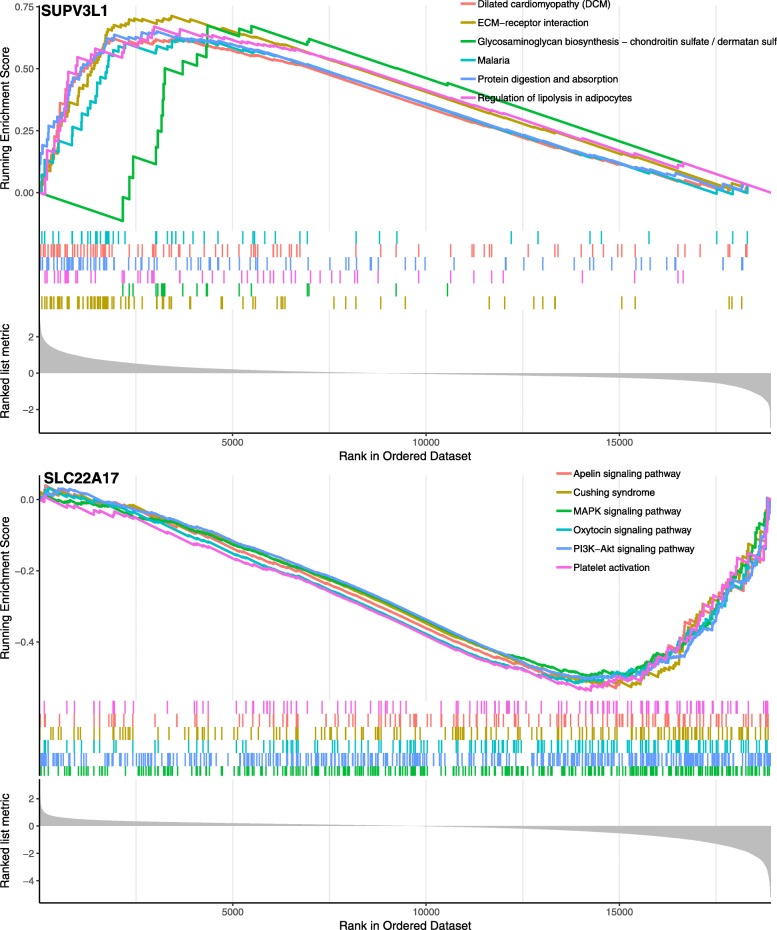
Gene set enrichment analysis (GSEA). Only listed the top six enrichment score gene sets

**Fig. 5:**
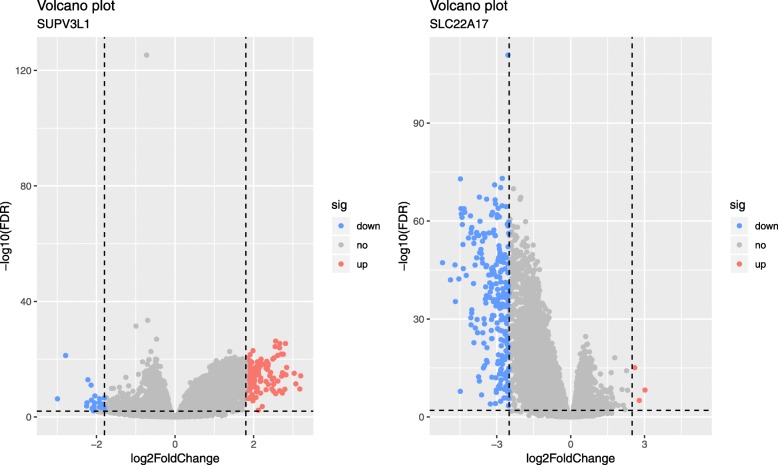
Volcano plot of the differentially expressed genes of SUPV3L1. Red indicates DEGs with log2foldchange > 1.8, *P* < 0.01, green indicates DEGs with log2foldchange < − 1.8, *P* < 0.01. Volcano plot of the differentially expressed genes of SLC22A17. Red indicates DEGs with log2foldchange > 2.5, *P* < 0.01, green indicates DEGs with log2foldchange < − 2.5, *P* < 0.01

**Fig. 6:**
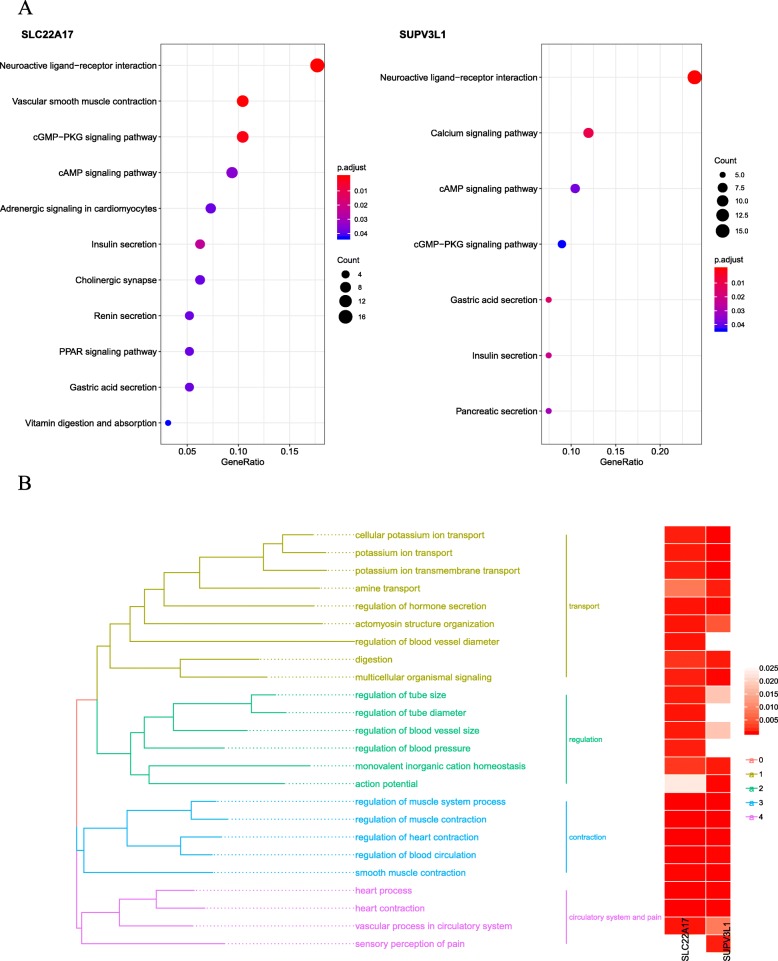
**a** KEGG analysis were used to obtain significant enriched KEGG terms. **b** Gene Ontology analysis were used to obtain significant enriched biological process (BP) terms, biological process of two genes are grouped according to functional theme

According to the groups of expression of SUPV3L1 and SLC22A17, difference analysis was used to investigate the biological role of SUPV3L1 and SLC22A17. To obtain further insight into the function of the hub gene, GSEA was conducted by “clusterProfiler” R package. Six representative pathways about SUPV3L1 were “Dilated cardiomyopathy (DCM)”, “ECM-receptor interaction”, “Glycosaminoglycan biosynthesis-chondroitin sulfate/dermatan sulfate”, “Malaria”, “Protein digestion and absorption” and “Regulation of lipolysis in adipocytes”, Six representative pathways about SLC22A17 were “Apelin signaling pathway”, “Cushing syndrome”, “MAPK signaling pathway”, “Oxytocin signaling pathway”, “PI3K-Akt signaling pathway” and “Platelet activation” (Fig. 4). 3 up-regulated and 236 down-regulated genes (| log2foldchange | > 2.5, *P* < 0.01) were identified significantly associated with SLC22A17 expression (Fig. 5), 125 up-regulated and 30 down-regulated genes (| log2foldchange | > 1.8, *P* < 0.01) were identified significantly associated with SUPV3L1 expression (Fig. 5). In order to explore biological relevance of differential genes, significantly differentially expressed genes were enriched using “clusterProfiler” R package for Gene Ontology (GO) and Kyoto Encyclopedia of Genes and Genomes (KEGG) analyses. Biological processes were grouped according to functional theme, it suggested to focus on “transport”, “regulation”, “contraction” and “circulatory system and pain” (Fig. 6a), KEGG analyses showed that hub genes may be related to “insulin secretion”, “cGMP-PKG signaling pathway”, “cAMP signaling pathway”, “calcium signaling pathway” and “neuroactive ligand-receptor interaction” (Fig. 6b).

## Discussion

The clinical success of immune checkpoint therapy recently, demonstrate the enormous potential of immunotherapy in cancer treatment. Currently, the main treatment method of patients with cancer is blocking CTLA-4 and PD-1 pathways and CAR T cell therapy. These methods were dependent on a sequence of basic science discoveries [16], Dong H et al. found out that antibodies blocking the PD-L1/PD-1 interaction lead to tumor regression in mice [17], transduced T cells of chronic lymphocytic leukemia patients can effectively lyse autologous tumor cells [18]. All these discoveries are based on research on immune cells and genes. Advances in next-generation sequencing permit the rapid research progress of mutant tumor neoantigens [8]. This article presented research linking gastric cancer to immune cells based on data of sequencing, thus deepened the understanding of the immune mechanism of gastric cancer.

Single-sample gene set enrichment analysis (ssGSEA) can be used to quantify immune infiltrating cell types based on the marker genes of immune cells [8, 10], based on RNA-seq data of Stomach adenocarcinoma (STAD) of TCGA and gene expression profile of GSE84437, ssGSEA was used to quantify immune infiltrating cell types of stomach adenocarcinoma samples. 24 kinds of tumor-infiltrating immune cells were quantified, and 3 kinds of immune cells (T helper type 2 (Th2) cells, T helper cells, and Mast cells) were identified as prognostic immune cells.

Through the analysis, Th2 cells and T helper cells were identified as protective factors, and Mast cells as a risk factor, but immune cells may play a dual role in cancers, even one kind of immune cells has a dual role. It has reported that Th2 cells can be used to eradicate cancer [19], and Th2 cells may promoting the immune escape of urological tumor [20]. T helper cells influence tumor antigen-specific ca cytotoxic T cell (CTL) response by producing many factors and further induce antitumor immunity [21]. Mast cells have the ability to facilitate tumor proliferation and invasion directly,and indirectly promote tumor proliferation and invasion by regulating tumor microenvironment [22], it may provide further evidence for Mast cells can be applied in the adjuvant treatment of mammary adenocarcinoma and melanoma [23]. Previous studies have shown that Th2 cells, T helper cells, and Mast cells may play key roles in the development and invasion of cancer [24–27], our studies show that these immune cells may play a role in gastric cancer.

However, the concrete mechanism is still unknown, further analysis was performed and two related hub genes (SUPV3L1 and SLC22A17) in three immune cells types of TCGA and GEO groups were regarded as hub genes for further validation, indicating that the two hub genes had a high connection with infiltration as well as prognosis. It has been reported that overexpression of SLC22A17 associated with poor prognosis of cancer, such as endometrial carcinoma, gliomas and hepatocellular [28–30], and Lipocalin-2 (LCN2) has the potential to alter immune cell infiltration and the tumor microenvironment in pancreatic ductal adenocarcinoma by downregulation of LCN2-specific receptor SLC22A17 [31]. These all indicate that SLC22A17 may influence prognosis through influencing immune cell infiltration and provided further evidence that SLC22A17 may play the same role in gastric cancer. But, research about SUPV3L1 on tumors is limited and further study is needed.

Below, we illustrated the differences between hub genes and prognostic immune cells in non-tumor tissues and tumor tissues within the context of specific gastrointestinal tumors. We can see the different infiltration of 3 kinds immune cells in normal and tumor tissues, Mast cells is less in tumor tissue, and Th2 cells is more in tumor tissue, it further suggested that immune cell infiltration may related to gastrointestinal tumors.

## Conclusion

In this paper, we found that three immune cells infiltration are associated with the prognosis of gastric cancer and further identify two hub genes. These two key genes may affect immune cell infiltration, result in the different prognosis of patients.

## Data Availability

The data of this study are from TCGA, GEO, GTEx and Kaplan Meier-plotter database.

## Abbreviations

DEGs: Differentially expressed genes
GEO: Gene expression omnibus
GO: Gene Ontology
GSEA: Gene set enrichment analysis
GTEx: Genotype tissue expression
HR: Hazard ratio
KEGG: Kyoto Encyclopedia of Genes and Genomes
SLC22A17: Solute carrier family 22 member 17
ssGSEA: single-sample Gene set enrichment analysis
SUPV3L1: Suv3 like RNA helicase
TCGA: The Cancer Genome Atlas
TMB: Tumor Mutation Burden
